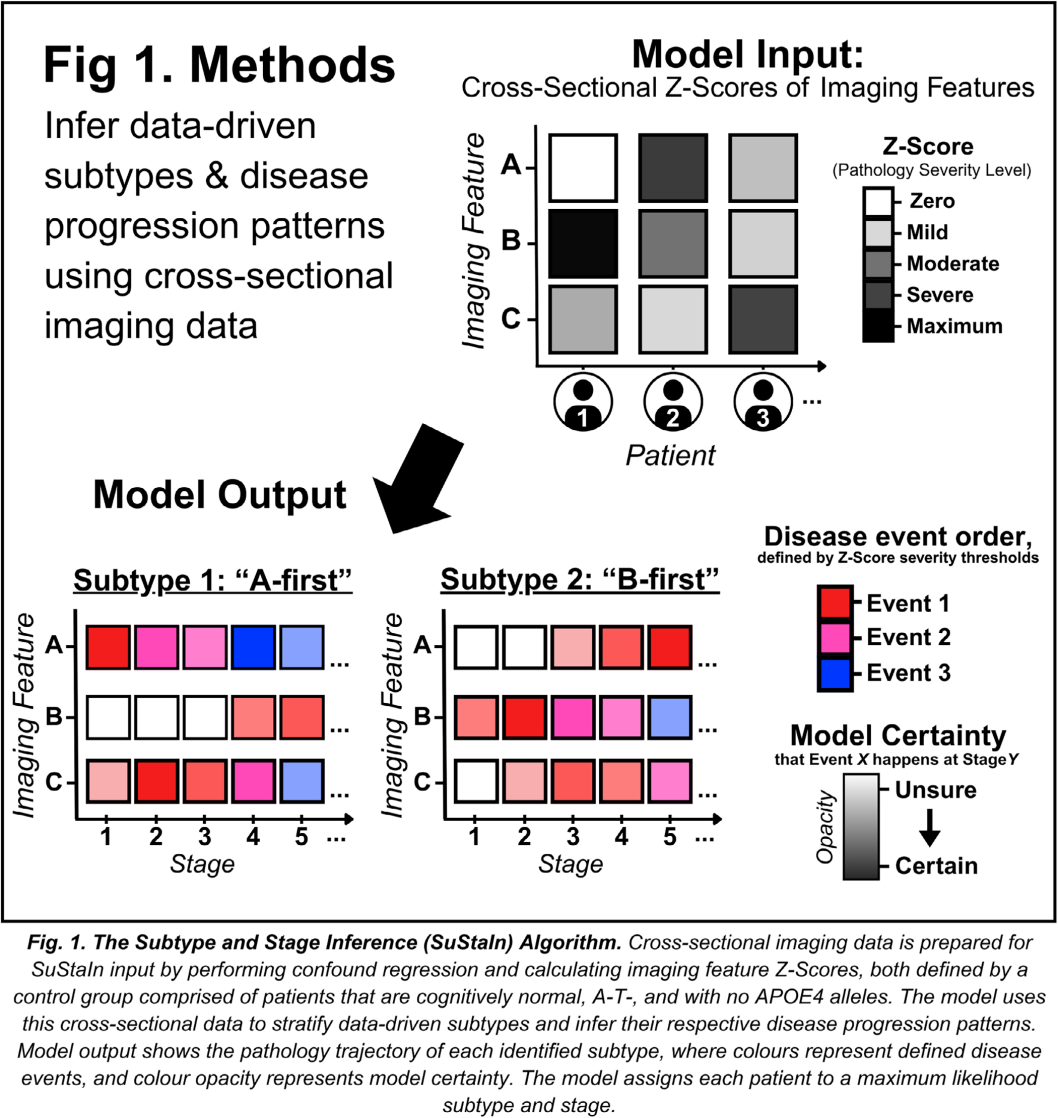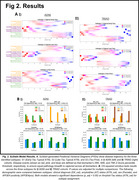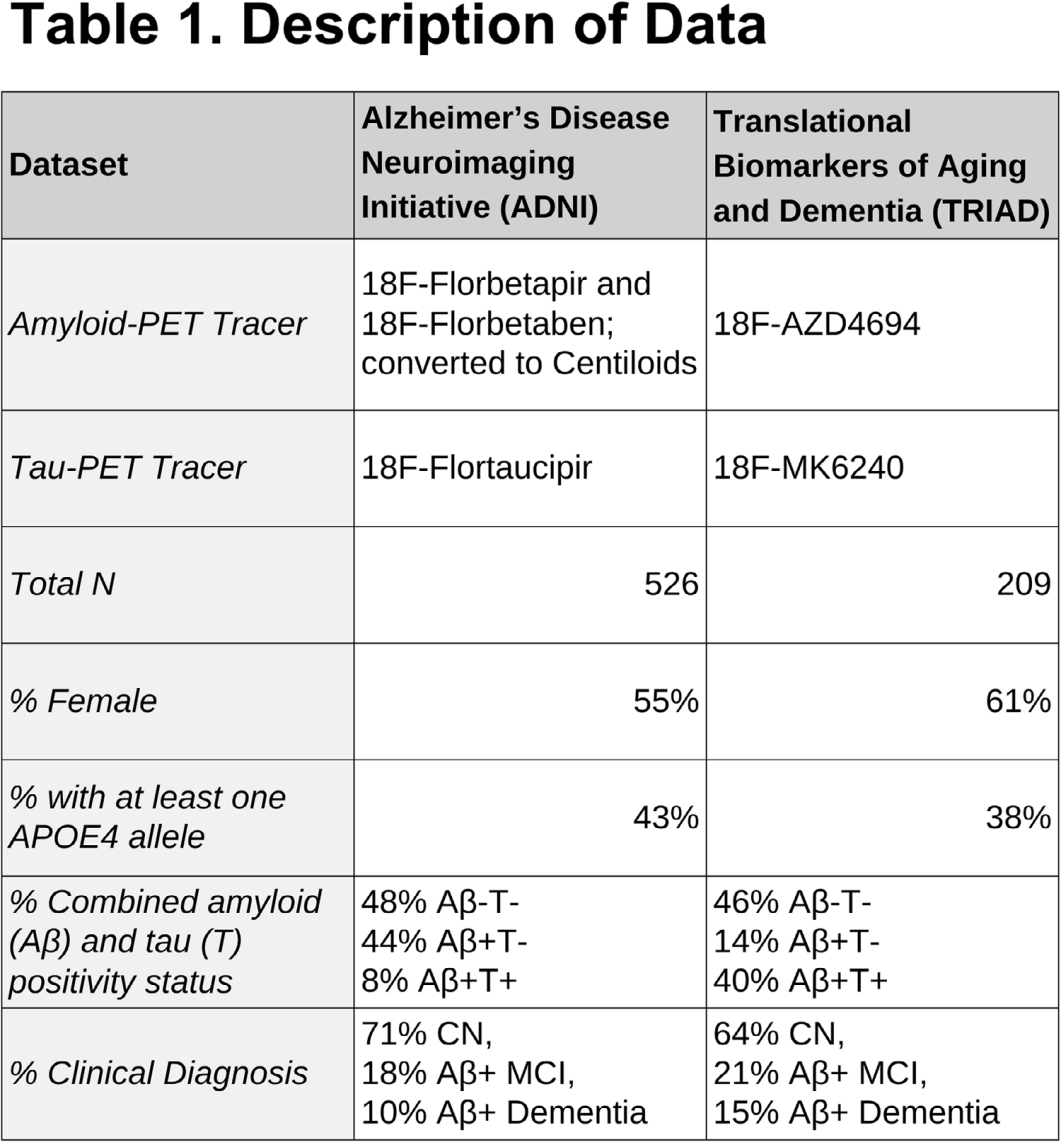# Identifying multimodal amyloid, tau, and neurodegeneration (ATN) subtypes in Alzheimer’s Disease across datasets and imaging markers

**DOI:** 10.1002/alz70861_108074

**Published:** 2025-12-23

**Authors:** Katrina Carver, Andrew Clappison, Min Su Kang, Nesrine Rahmouni, Jenna Stevenson, Walter Swardfager, Joanne Mclaurin, Bojana Stefanovic, Richard H. Swartz, Sean M. Nestor, Jennifer S Rabin, Serge Gauthier, Jean‐Paul Soucy, Jean Chen, Mario Masellis, Sandra E. Black, Pedro Rosa‐Neto, Julie Ottoy, Maged Goubran

**Affiliations:** ^1^ Artificial Intelligence and Computational Neurosciences Lab, Sunnybrook Research Institute, University of Toronto, Toronto, ON Canada; ^2^ Translational Neuroimaging Laboratory, The McGill University Research Centre for Studies in Aging, Montréal, QC Canada; ^3^ Hurvitz Brain Sciences Program, Sunnybrook Research Institute, Toronto, ON Canada; ^4^ Sunnybrook Research Institute, Toronto, ON Canada; ^5^ Division of Neurology, Department of Medicine, Sunnybrook Health Sciences Centre, Toronto, ON Canada; ^6^ Hurvitz Brain Sciences Research Program, Sunnybrook Research Institute, Canada, Toronto, ON Canada; ^7^ Montreal Neurological Institute, McGill University, Montréal, QC Canada; ^8^ Rotman Research Institute, Baycrest, University of Toronto, Toronto, ON Canada; ^9^ Hurvitz Brain Sciences Program, Toronto, ON Canada; ^10^ Montreal Neurological Institute, Montréal, QC Canada; ^11^ Dr. Sandra E. Black Centre for Brain Resilience and Recovery, Toronto, ON Canada

## Abstract

**Background:**

Alzheimer’s Disease (AD) is heterogeneous in pathology and clinical presentation. A comprehensive study on the spatio‐temporal progression of multimodal imaging biomarkers in AD is currently lacking. Here, we identified multimodal subtypes of amyloid, tau, and neurodegeneration (ATN) progression in two independent datasets across the AD spectrum.

**Method:**

We employed cross‐sectional datasets from the ADNI (376 CN, 95 Aβ+ MCI, and 55 Aβ+ Dementia) and TRIAD (133 CN, 45 Aβ+ MCI, and 31 Aβ+ AD) studies (Table 1). We applied the Subtype and Stage Inference (SuStaIn) algorithm, which accounts for phenotypical and temporal heterogeneity, to identify subtypes (Figure 1). The following multimodal imaging markers were used in SuStaIn: 18F‐AZD4694 amyloid‐SUVR or 18F‐Florbetapir and 18F‐Florbetaben Centiloids, 18F‐MK6240 or 18F‐Flortaucipir tau‐SUVR, and T1‐weighted MRI volumes. Each marker was evaluated in four meta‐ROIs: medial temporal (MTL), lateral temporal, frontal, and parieto‐occipital. We studied the disease progression patterns of each generated multimodal subtype and its demographic features using omnibus and post‐hoc statistics.

**Result:**

Both cohorts identified three subtypes: “early‐tau typical ATN”, “late‐tau typical ATN”, and “tau‐first” (Figure 2). The “early‐tau typical ATN” subtype showed initial increases in frontal and neocortical Aβ, followed by MTL and neocortical tau, and eventually widespread atrophy. The “late‐tau typical ATN” subtype, on the other hand, showed the earliest increases in entorhinal‐hippocampal Aβ, followed by severe widespread Aβ, with frontal being the latest, and eventually neocortical tau with atrophy. Finally, the “tau‐first” subtype’s progression was similar to the “early‐tau typical ATN” subtype with an additional early tau wave, potentially indicating primary age‐related tauopathy (PART) pathology that may or may not transition into AD. We observed a statistically significant dependent relationship between subtype membership and AT status (*p* _adj < 0.05). The “tau‐first” subtype was significantly younger than other subtypes (*p* _adj < 0.05).

**Conclusion:**

Our findings replicated the previously reported typical amyloid‐first (ATN) subtype and tau‐first subtype, but demonstrated that the typical amyloid‐first subtype in fact consists of an early‐ and a late‐tau subtype. These insights into the progression patterns of A, T, and N imaging biomarkers across datasets and radioligands may inform trials and understanding the heterogeneity of pathology trajectories.